# A mini review of leveraging biobanking in the identification of novel biomarkers in neurological disorders: insights from a rapid single-cell sequencing pipeline

**DOI:** 10.3389/fnins.2024.1473917

**Published:** 2024-12-24

**Authors:** Joseph S. Miller, Michael Rose, Jonathan Roell, Samruddhi Ubhe, Tom Liu, Benjamin M. Segal, Erica H. Bell

**Affiliations:** ^1^Heritage College of Osteopathic Medicine, Ohio University, Dublin, OH, United States; ^2^Department of Neurology, College of Medicine, The Ohio State University, Columbus, OH, United States; ^3^Neuroscience Research Institute, College of Medicine, The Ohio State University, Columbus, OH, United States

**Keywords:** biobanking and biorepositories, biospecimens, neurologic biomarkers, next-generation sequencing, single-cell sequencing

## Abstract

Recent successes in the identification of biomarkers and therapeutic targets for diagnosing and managing neurological diseases underscore the critical need for cutting-edge biobanks in the conduct of high-caliber translational neuroscience research. Biobanks dedicated to neurological disorders are particularly timely, given the increasing prevalence of neurological disability among the rising aging population. Translational research focusing on disorders of the central nervous system (CNS) poses distinct challenges due to the limited accessibility of CNS tissue pre-mortem. Nevertheless, technological breakthroughs, including single-cell and single-nucleus methodologies, offer unprecedented insights into CNS pathophysiology using minimal input such as cerebrospinal fluid (CSF) cells and brain biopsies. Moreover, assays designed to detect factors that are released by CNS resident cells and diffuse into the CSF and/or bloodstream (such as neurofilament light chain [NfL], glial fibrillar acidic protein [GFAP] and amyloid beta peptides), and systemic factors that cross the blood–brain barrier to target CNS-specific molecules (e.g., autoantibodies that bind either the NMDA receptor [NMDAR] or myelin oligodendrocyte glycoprotein [MOG]), are increasingly deployed in clinical research and practice. This review provides an overview of current biobanking practices in neurological disorders and discusses ongoing challenges to biomarker discovery. Additionally, it outlines a rapid consenting and processing pipeline ensuring fresh paired blood and CSF specimens for single-cell sequencing that might more accurately reflect *in vivo* pathways. In summary, augmenting biobank rigor and establishing innovative research pipelines using patient samples will undoubtedly accelerate biomarker discovery in neurological disorders.

## 1 Introduction

### 1.1 Biobanking in neurology

Biobanks play a pivotal role in advancing neurological research, translating wet bench findings into clinical applications, and catalyzing precision medicine in Neurology. Neurological disorders are a leading cause of mortality and morbidity, imposing significant social and economic costs on patients, families, and the healthcare system (Feigin et al., 2020). The burden of neurological disease is expected to increase globally as the human population expands and ages. Robust, comprehensive biobanks focused on Alzheimer’s disease (AD) have been established in academic medical institutions for years; however, protocols for specimen collection, processing and storage, as well as the scope and quality of associated clinical databases, vary between institutions. Moreover, systematic biobanking of specimens from individuals with other neurological conditions is relatively limited.

In many neurological disorders pathological changes begin silently, years before clinical presentation (Jack et al., 2018). In some instances, this prodromal period may be accompanied by altered biomarker expression (Jack et al., 2018; Achiron et al., 2010). For example, serum NfL levels and amyloid beta peptide levels are elevated from baseline several years prior to the clinical presentation of multiple sclerosis (MS) and AD, respectively (Bjornevik et al., 2020; Stocker et al., 2020). Developing neurology-specific longitudinal biobanks that include specimens from individuals who are statistically at high risk for future development of neurological diseases (e.g., identical twins and first-degree relatives of patients, carriers of known genetic risk loci, etc.) will be essential for elucidating prodromal biomarkers, enabling earlier diagnosis and intervention. Of equal importance is the identification of biomarkers that are predictive of therapeutic responses to specific disease modifying therapies or their side effects, a cornerstone of precision medicine. Examples in Neurology include anti-JC virus antibody titers that help stratify the risk of progressive multifocal leukoencephalopathy (PML) in MS patients following the initiation of natalizumab or fingolimod (Sgarlata et al., 2022). Furthermore, natalizumab-induced lymphocytosis and ocrelizumab-induced B cell depletion are indicative of drug efficacy, whereas the appearance of neutralizing antibodies against beta-interferon or natalizumab correlate with loss of efficacy (Signoriello et al., 2016; Vennegoor et al., 2013; Sorensen et al., 2003).

Although limited in numbers, there are multiple examples of cutting-edge CNS-focused biorepositories including the NIH NeuroBiobank, The Arizona Study of Aging and Neurodegenerative Disorder, the Biorepository at the Barrow Neurological Institute, and BRAINUK. The NIH NeuroBioBank (started in 2013) is now a consortium of six biorepositories that prioritizes the collection of post-mortem brain tissue, blood, CSF, skin, and samples from other organ systems to facilitate a comprehensive biobank that researchers can utilize to investigate various neuropathology (Freund et al., 2018). The Arizona Study of Aging and Neurodegenerative Disorders historically collected brain and CNS tissue, serum and plasma, and scalp and head muscle biopsies; however, starting in 2005, the biobank began collecting bodily tissues from most organ systems due to the increased understanding of the significant interactions between the brain and the body (Beach et al., 2015). Further, leading neurologic biobanks such as the Barrow Neurological Institute’s biobank emphasize the collection of freshly frozen tissue, as well as matched blood components (plasma, serum, PBMCs, and whole blood) and CSF. The biobank also provides RNA, DNA, and protein concentration and quality analysis for investigators (Seiler et al., 2015). An adjunct to the Barrow Institute’s biobank is their provision of flow cytometry and genomics core which can provide transcriptomic analysis and RNA sequencing for interested investigators.

Whereas, BRAIN UK prioritizes tissue samples with biopsies of the brain, muscle, peripheral nerves, ophthalmologic specimens, as well as CSF cytology (Nicoll et al., 2022).

Although there are substantial needs for CNS-specific biobanks, there are many limitations with procuring CNS-related tissue. For example, these include limited accessibility and delicacy of CNS tissues and the high cost of procuring quality post-mortem brain tissue. In regard to the former, a key logistic hurdle is minimizing the time interval between death and specimen procurement in order to limit RNA and protein denaturation (Aquila et al., 2018; Zhang et al., 2017). Further, post-mortem tissue only provides a “snapshot” at the end of the disease process. The development of blood- or CSF-based biomarkers that correlate with neuropathological activity represent a distinct advantage by allowing insights into the diagnosis, evolution and therapeutic responsiveness of neurological diseases in living patients. This goal can only be achieved by routine longitudinal, as well as cross-sectional, collection of sera, plasma, peripheral blood mononuclear cells (PBMC), CSF and CSF cells, from well characterized subjects with a range of neurological disorders, in conjunction with high quality clinical and paraclinical outcome measures (Blokker et al., 2017; Nemetz et al., 2006). Many neuroscience-specific biobanks have historically collected post-mortem tissue, blood (including plasma/serum), and CSF when available. At The Ohio State University, we have begun to routinely collect PBMCs on all neurological patients. In addition, we are increasing our collection of skin, muscle, and nerve biopsies from patients harboring neurological disorders. The collection of skin biopsies enables high-quality production of fibroblasts and iPSCs for research (Mommaerts et al., 2022). Importantly, we would advocate that biospecimens be routinely collected pre- and post-introduction of novel disease modifying therapies during clinical trials in order to perform mechanistic sub-studies and identify predictive and surrogate markers of therapeutic responsiveness. CSF and CSF cells may be especially valuable due to their proximity to, and interaction with, CNS resident cells. In this review, we will outline the unique aspects of biobank development and standard biobanking practices. We will also discuss a unique pipeline for optimizing the collection of samples for single-cell sequencing toward biomarkers of neurologic diseases.

### 1.2 Neurologic biobank development and optimization

Biobank development is an extensive undertaking, posing challenges with respect to logistics, consistency of biospecimen integrity, and data security (Annaratone et al., 2021). Frequently, resources necessary to support biobank creation and maintenance are limited. This is further compounded by the lack of standardized nomenclature, universal protocols for biospecimen collection, processing, storage and cataloging, and guidelines for database acquisition and management (Annaratone et al., 2021). The development of protocols for biospecimen handling is particularly important because poor-quality specimens may result in false discovery and insufficient specificity/sensitivity to be used as clinical tools (Schully et al., 2015). Additionally, without universal standards for biobanking in neurological diseases, the opportunity for institutional collaboration is curtailed (Hanash, 2011; Poste, 2012). Limited specimen availability, especially for longitudinal collection pre- and post-treatment, impedes the identification of surrogate biomarkers and the validation of initial research findings with independent cohorts (Batis et al., 2021; Taube et al., 2009).

The development of a versatile and trustworthy biobank-associated database may be undermined by variations in the quality, scope and reliability of demographic and clinical data collected by different healthcare providers, inaccurate diagnoses, inconsistencies in data storage systems and formats, and ethical/legal barriers to data sharing. High quality, reliable clinical data is critical for contextualizing samples based on patient demographics, medical, family and social history, co-morbidities, potentially confounding environmental factors, and treatment outcomes. A meticulous accounting of all of this information is essential for identifying and validating clinically useful biomarkers and therapeutic targets. A well-designed clinical database also facilitates data sharing across studies, which may enable identification of patterns not apparent in individual studies.

Biobanks are established to support biomedical research, help researchers study pathologies, develop biomarkers and establish their clinical utility, and identify therapeutic targets. At The Ohio State University, we have formed a committee within the Neuroscience Research Institute responsible for overseeing our biobank’s policies and practices, and to screen, review, and approve requests or require modifications for the release of biospecimens to ensure the scientific rigor of the study and specimen use. Requests for large sample sizes, large numbers of biospecimens, rare biospecimens, or biospecimens in high demand may be granted if the research is of sound scientific value, high importance, and where possible the use does not negatively impact the availability of biospecimens for other research interests. Members of the committee span 5 departments, including Neurology, Neurosurgery, Psychiatry and Psychology, and include wet bench and clinical scientists, as well as clinicians. Communication with all pertinent stakeholders about every aspect of biobank development, as outlined below, is critical for its successful execution:

1. **Ethical considerations:** Issues related to informed consent, privacy protections, return of clinically significant findings and confidentiality should be considered by the oversight committee, biobank manager and support staff, as well as users. Particularly for neuro-specific biobanks, the inclusion of cognitively-impaired participants adds another layer of regulatory complexity.
  - Biobank consenting should be forward thinking to ensure viability of specimen for the foreseeable future, abide by regulatory and institutional policies, and guarantee a truly informed consent process (Chandrashekar et al., 2022).
  - Given that biobank data are increasingly stored digitally, institutions should make all efforts to ensure their storage modalities are efficient and secure to prevent breach of participant data, as well as ensuring that samples can be easily traced to their respective donor by only the minimal research staff (Bukreeva et al., 2024).
  - The prevailing mindset surrounding return of clinically significant findings supports the return of results relevant to the participant’s health. Furthermore, the current literature supports the return of these results to family following the death of the participant, regardless of their consent. Therefore, biobank consent should work with their institutions to consider how to navigate this process and how to integrate this consideration into future research projects using collected biospecimens and their corresponding consent forms (Wolf, 2013).
2. **Community engagement:** Community leaders, patient groups, and advocacy organizations should have an opportunity to share valuable insights into the cultural, social, and political context of the biobank.
3. **Research outcomes:** Both researchers and funders have a personal stake in the quality and relevance of research outcomes.
4. **Governance and oversight:** Standardization and security in all aspects of sample collection, processing, distribution and storage are required.

#### 1.2.1 Standardization best practices

Biobanks are large repositories of patient- and disease-specific information and biospecimens, the contents of which are adaptable according to the institute’s unique research needs. Sufficient staffing of the biorepository is key including a manager(s) that oversees all aspects of regulatory requirements, consenting, specimen processing, and long-term storage including freezer temperature monitoring and a contingency plan for freezer failure. Although universal biobank protocols have yet to be widely adopted, multiple organizations, including the International Standardization Organization (ISO), have developed their own internal guidelines to ensure high-quality biobank performance and enable collaboration(BBMRI-ERIC, n.d.; European, Middle Eastern and African Society for Biopreservation and Biobanking, n.d.; ISBER, 2018; Standardization IOf, 2018; Cooperation OOfE, Development, 2010). The utilization of Standard Operating Procedures (SOPs) for every procedure is essential specifically for collections at specific-time points or logistically complex pipelines [see (Perry et al., 2019), (Holland et al., 2018), and (Teunissen et al., 2014) for a detailed review on whole blood/plasma/serum, PBMCs, and CSF processing, respectively]. These SOPs ensure reliable sample collection across diverse clinical settings by outlining sample processing techniques for plasma, serum, PBMCs, whole blood, and CSF (including volume, reagents, and products used to ensure consistency), guidelines for storage of samples and how affiliated sample information will be stored and maintained, and directives for unexpected events. Given clinical encounters are more frequently occurring at multiple sites, these SOPs also outline high-quality transportation of specimen to the main storage site, for example temperature monitoring of dry ice coolers to ensure samples are stable during transport to the main facility. Additionally, the SOPs provide regulatory information regarding protection of human subjects, responsibilities of investigators and personnel, and any institutional requirements of clinical research. These SOPs can be individualized to each unique institute and biobank. Data quality, consistency, and reliability across multiple sites is ensured by personnel compliance to SOPs. Importantly, the College of American Pathologists has established a biorepository accreditation program (BAP), through which qualifying biorepositories can receive a BAP certificate demonstrating adherence to best practices in biorepository management (McCall et al., 2018). The construction of a high-quality and forward-thinking biobank requires well-described processes conducted in alignment with standardization efforts (Table 1) (Cooperation OOfE, Development, 2010).

**Table 1:** Essential components in neuro-focused biobank construction.

Biobank components	Best practices in development
Core elements	Establish clear objectives for biobank, as well as legal and ethical framework. Develop standardized operating procedures and policies to ensure biobank transparency, consistency, participant confidentiality, accessibility, and procedures for communicating results.
Biobank establishment	Ensure adequate resources for efficient operation and sustainability. Consult relevant stakeholders and communicate biobank purpose, including methods to prioritize participant interests.
Governance	Create a well-documented structure of responsibility, governance, and oversight processes. Develop comprehensive policies to maintain standards in the event of investigator departure.
Participation	Outline guidelines for participant recruitment with prioritization of freedom of choice in participation with right to withdraw at any point. Develop guidelines to protect vulnerable populations and when substitute decision makers or waiver of consent may be applicable. Clarify when disclosure of identifiable information may be required. Explain possibility for commercial products and any compensation available.
Specimen and data	Ensure high-quality quality control measures in line with accepted standards at every stage of sample processing. Develop clear protocols to protect data and policies detailing if/when clinical data may be accessed and how this data may be associated with specimen. Detail methods for sample storage and distribution, as well as specimen nomenclature unique to each sample. Establish well-documented data management protocols and a capable laboratory information management system.
Protection protocols	Ensure governance’s and processes’ focus on comprehensive protection of participant information and biospecimen at all stages of research.
Sample utilization	Create well-documented protocol with collaboration of Institutional Review Boards for if/when researchers using samples may access identifiable information and ensuring adequate research plan precautions to prevent breach of patient privacy. Establish requirement for research plans that are scientifically sound and in line with participants’ informed consent. Detail how specimen requests will be prioritized and defined criteria for sample access.
Personnel	Explicitly communicate biobank goals and emphasis on participant privacy to personnel. Ensure the qualification and competency of biobank personnel with disclosure of conflicts of interest.
Good stewardship	Orient biobanking toward sharing of derived benefits. Explicitly communicate retained rights to participants. Develop clear policies outlining rights from intellectual property or commercialization derived from biospecimen research.
Biobank termination	Establish protocol detailing how biospecimens and data will be managed in the event that biobank material will be destroyed with emphasis on the protection of patient privacy.
CNS-specific biobanking	Create protocol for consenting of cognitively-impaired patients and utilization of a legally authorized representative (if applicable). Determine site-specific CNS biobanking needs (e.g., post-mortem tissue and/or CSF). Establish communication and pipeline with local morgue and neuropathology for post-mortem procurements. Develop standard operating procedures for specialized tissue needs to ensure quality of specimen (ex. cryopreservation temperature and storage media (CSF) or post-mortem interval time for autopsy tissue).

#### 1.2.2 Impact of standardization

By following best practices, biobanks will be able to:

1. Minimize physical and data breach risks to the participant.
2. Minimize ethical and legal risks related to the biorepository.
3. Increase researcher and public confidence in biobanks.
4. Improve biospecimen quality and research.
5. Streamline sample processing and storage efficiency.
6. Share specimens across biobanks and study a generalizable population.
7. Enhance data reliability.
8. Successfully perform sensitive laboratory procedures in-house.

Overall, a standardized approach optimizes researchers’ efforts, fosters collaboration, and positions institutes/hospitals as leaders in this rapidly developing research area of neurological-specific biomarkers.

### 1.3 Neurologic biomarkers

Neurological biomarker research is exponentially expanding, driving the need for high-quality biobanks (Lleó, 2021). CNS-specific serological and CSF biomarkers are proving useful in establishing diagnoses and formulating prognoses at different stages of disease and, in some cases, heralding the prodromal period (Dubois et al., 2016; Frisoni et al., 2017). Analyses of paired CSF and blood samples can be particularly impactful by revealing CNS-specific phenomena. Hence, some biomarkers are exclusively found in CSF while others are enriched in the CSF. A classic example of the former is the presence of unique oligoclonal bands (OCBs) in the CSF but not the serum (representing monoclonal antibodies only produced in the CNS) in individuals with MS and other neuroinflammatory disorders. OCBs are a component of the McDonald consensus criteria for the diagnosis of MS (Thompson et al., 2018). Certain CSF markers, such as CSF amyloid-β (Aβ_42_) levels, are among the earliest indication of CNS pathology (Zou et al., 2020). Other core CSF biomarkers including total tau, phosphorylated tau, and Aβ42 facilitate diagnosis of AD early in the clinical course (McGrowder et al., 2021). Elevated CSF levels of inflammatory markers, such as cytokines, are more sensitive and specific indicators of neuroinflammation than their levels in paired serum levels (Gigase et al., 2023). Measuring biomarker levels in paired blood and CSF can also increase the accuracy of diagnosis in instances where molecules, such as tau, are processed differently systematically versus centrally (Gaetani et al., 2020).

Blood-based biomarkers are logistically advantageous because they are less invasive, have fewer side effects, and facilitate monitoring and screening at routine visits (Teunissen et al., 2018). However, some biomarkers may be more accurate in either the CSF or blood. For example, CSF autoantibodies are at the cornerstone of diagnosis for NMDAR encephalitis (due to the increased concentration in CSF) (Gresa-Arribas et al., 2014), whereas for MOG antibody-associated disease blood autoantibodies are first line diagnostic testing (Matsumoto et al., 2023). Serologic biomarkers that have been reported to reflect neuropathological processes include CCL23 in acute ischemic stroke, phosphorylated tau and amyloid-β in AD, and NfL levels in relapsing–remitting MS (Ashton et al., 2021; Kuhle et al., 2019; Simats et al., 2018). Another example can be found within mild traumatic brain injury (TBI), where the investigators showed via a meta-analysis that children with elevated serum protein S100B, a calcium-binding protein and known biomarker of brain injury, levels correlated with the presence of intracerebral lesions, as demonstrated via CAT scan (Oris et al., 2018). Additionally, plasma GFAP and plasma tau proteins are elevated in adult patients with acute and chronic TBI, further demonstrating the utility of molecular techniques as a complementary diagnostic tool (Feng and Jiang, 2020; Abdelhak et al., 2022). Proteomic and transcriptomic signatures of PBMCs may be altered in the context of neurological diseases. Transcripts encoding the RNA binding protein TARDP3 (TDP-43) are enriched in PBMCs from people with ALS, a consequence of the dysregulated RNA metabolism that has emerged as a central process in disease etiology (Pansarasa et al., 2022). Specifically, PBMCs may reflect pathologic CNS findings in neurodegenerative diseases and demonstrate protein synthesis variations across other conditions such as cancer or autoimmune disorders, supporting their use for biomarker identification with less risk to the patient (Arosio et al., 2014; Bian et al., 2014; Neilson et al., 2020; Xiong et al., 2021; Alexovic et al., 2024). In summary, biomarkers serve as invaluable tools in novel therapeutic development and the monitoring of individual treatment responses (Group et al., 2001).

### 1.4 Promise of new technologies: single cell sequencing at the forefront

Microarray and next generation sequencing (NGS) techniques have advanced researchers’ ability to assess genomic pathogenicity in neurologic conditions. Of note, NGS was recently used to create a prediction model using cell-free plasma miRNA that differentiated frontotemporal dementia from controls with 90% accuracy (Magen et al., 2023). Similar studies have used cell-free plasma messenger and microRNAs to differentiate AD from controls (Magen et al., 2023; Toden et al., 2020). Single cell RNA sequencing (scRNA-seq) is increasingly used by researchers to gain insights into cellular and molecular mechanisms in the context of neurological diseases. Historically, the role of T and B cells in neurological conditions has been difficult to study due to polyclonality, but scRNA-seq has enabled researchers to examine the role that antigen-driven clonal expansion and lymphocyte polarization plays in the pathogenesis of neuroimmunological diseases such as MS and autoimmune encephalitis (Gaublomme et al., 2015). This technique has also been informative in distinguishing disease-relevant antibodies from irrelevant ones (Spadaro et al., 2018; Schafflick et al., 2020). Intriguingly, scRNA-seq has revealed altered TCR and BCR repertoires in the blood of patients with AD (Xu and Jia, 2021).

### 1.5 Leveraging biobanks to identify novel innovative neurological biomarkers

A high-quality, standardized biobank can address biomarker development barriers by providing ample specimens consistent in collection and processing harmonized with clinical data. Academic centers are working to ensure biomarker study standardization, adequate powering, and reproducibility, as well as the implementation of protocols for processing, analysis, and validation (Andreasson et al., 2015; Del Campo et al., 2012; Otto et al., 2012).

One advantage of efficient biobanking structures is that integrated biobanking protocols can provide rapid access to fresh specimens, thus maintaining physiologic and biochemical properties. Fresh processing is essential, especially in specimens that are sparse or difficult to access (e.g., CSF white blood cells). Using fresh biospecimens (similar to an *in vivo* state) for transcriptomic analyses is optimal given that transcriptional changes have been identified in peripheral white blood cells within 8 h of refrigeration, which may alter research findings (Wilson et al., 2022). For example, a rapid sequencing pipeline for subjecting fresh specimens to scRNA-seq preserves cellular and microenvironment integrity and enables examination at the individual cell level. This approach couples biomarkers with the current disease state and may identify biomarkers that enable early diagnosis and monitoring of therapeutic response.

**Figure 1:**
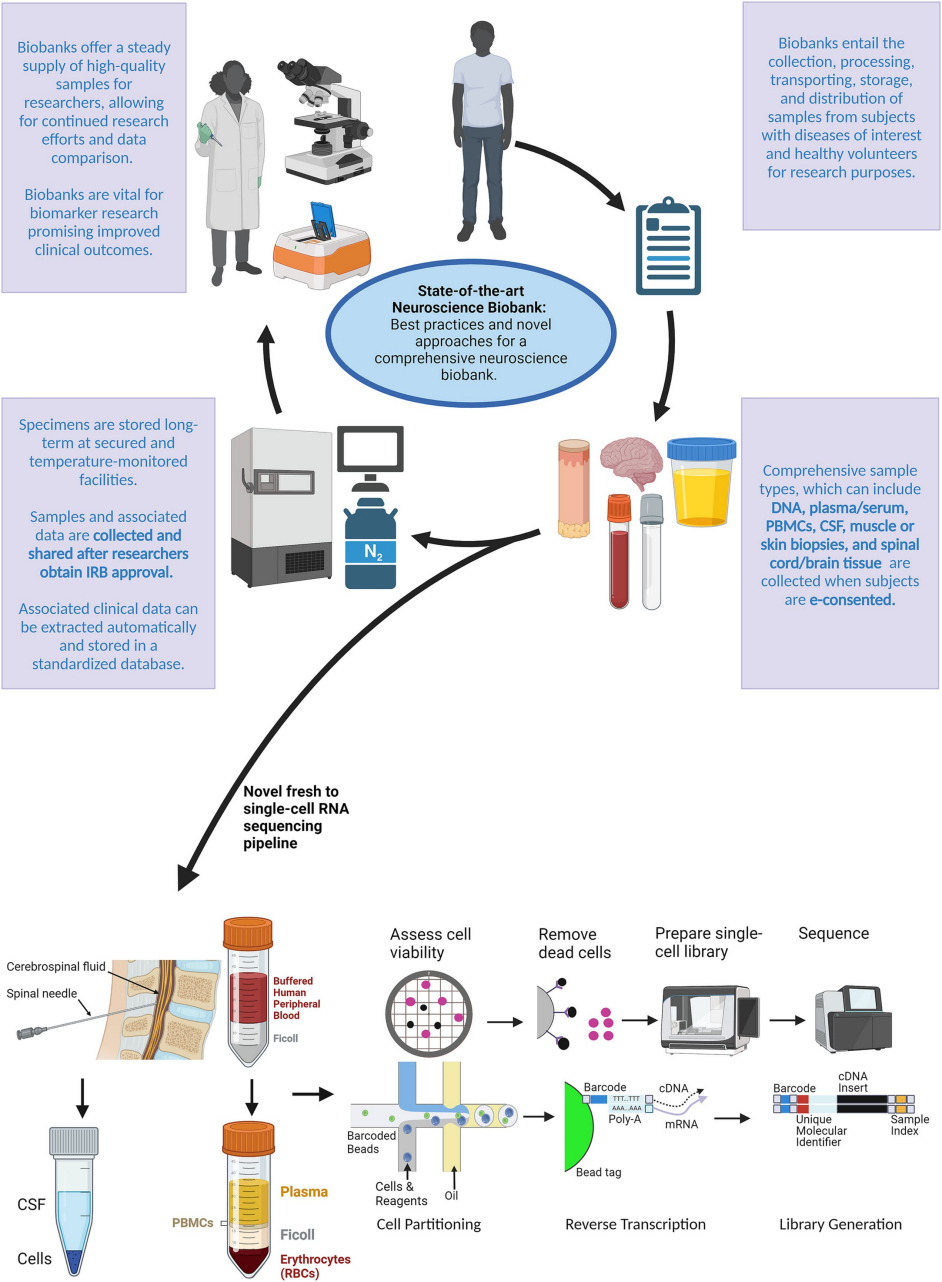
Schematic outlining a novel approach for a comprehensive neuroscience biobank. After biobank creation, subjects with the disease(s) of interest and healthy volunteers can be electronically consented to the study protocol during routine or emergency visits. After being consented to the study protocol, specimens can be collected according to the desired tissue type outlined in the IRB approved protocol. Traditionally, samples and associated data are then stored at on-site facilities. We introduced a novel pipeline for processing fresh specimens using single-cell RNA sequencing. Researchers can then use these specimens and associated data toward continued research efforts to identify and develop biomarkers that may improve clinical outcomes. Created with BioRender.com.

To explore the potential power of combining a robust sample collection pipeline and rapid sequencing, our team implemented rapid single-cell library preparations from fresh PBMC and CSF cells (Figure 1). Samples were collected in the inpatient and outpatient clinics, delivered to a centralized laboratory on the same day, and processed within hours. Samples from patients with acute neurological presentations were targeted, which introduced new challenges. First, these patients required urgent medical attention, complicating consenting and sample collection prior to a standard of care lumbar puncture and/or treatment administration. Second, neurological emergencies may present outside of normal work hours limiting the availability of trained personnel. The workflow requires:

1. **Consistent training:** Ensure adequate personnel who are appropriately trained in consenting, processing, and logistical procedures to minimize missed enrollments and specimen degradation.
2. **Streamlined consenting:** Maintain a database that enables real-time visualization and notification of enrollment in the study as specimens are collected across multiple sites. This prevents dual assignment of study-ID number and allows the lab team to anticipate specimens. Electronic consent streamlines consent tracking and efficiency with data management and storage.
3. **Robust communication:** High quality group communication delivered at specific process timepoints following established routes and organization between the Emergency Medicine, Neurology, and biobank team is integral to coordinating same day consent, collection, and specimen processing. Standardized chain of custody workflows and process visualizations including labels, signs and diagrams in designated pick-up and drop-off areas are critical.
4. **Standardized protocols:** Establish standard protocols for consenting, sample pickup, and sample processing that outlines the type of sample, required equipment and materials, and processing steps, limiting potential sources of variation.
5. **Timely transport:** Have supplies readily available and well-established communication points to facilitate transport of samples to the lab in a regulated and timely manner to prevent degradation or loss. Temperature monitoring in both temporary and long-term storage should also be utilized to preserve sample integrity, allowing fast action in events of an excursion.
6. **Quality control:** The use of appropriate controls and standards should be incorporated to ensure the accuracy and consistency of results. Specimens should be tracked via a sample intake process notating the sample type, amount, collection time, processing time, staff involved, and any quality control items such as clotted blood, cracked tubes, or lower volume collections.

This rapid workflow has efficiently and effectively produced high-quality samples for research purposes, even during off-hours. The pipeline has enabled timely analysis while limiting the possibility for sample degradation, thereby preserving *in vivo* cell signatures. Finally, the pipeline has resulted in library and sequencing completion in less than 2 weeks under most conditions.

By optimizing sample collection and processing through collaboration with attending physicians, fellows, residents and clinical trial coordinators, the research team was able to perform timely analysis and preserve sample integrity. The key to this success was the robust infrastructure of a forward-thinking comprehensive neuroscience biobank that facilitated patient consent and specimen collection, and provided a technical staff trained in specialized library preparation (ex. single cells). Recent literature has demonstrated the utility of single-cell technologies in analyzing the expression patterns of risk genes for neurological disorders, thereby allowing researchers to identify temporal patterns in various neurologic cell lineages (Kim et al., 2024). Furthermore, single-nucleus RNA sequencing techniques have demonstrated distinct gene expression and dysregulations within the neuronal cells of patients with epilepsy, dementia, and neurologic tumors with variation based on donor characteristics, disease states, and genetic regulation (Johansen et al., 2023). These studies support the use of single cell and nucleus approaches for the development of comprehensive data pertaining to neurologic diseases. Researchers affiliated with UK Biobank have conducted single cell and epigenetic studies in patients with multiple sclerosis which allowed for the identification of susceptibility genes that may guide therapeutic targets for MS therapeutics (Ma et al., 2023). Other UK Biobank affiliated researchers utilized scRNA-seq to support the role of *EGFR* in Alzheimer’s disease pathology and characterize the context of *EGFR* signaling that could be utilized for developing future targeted therapeutics for Alzheimer’s disease (He et al., 2021). Developing and investing in neuro-focused biobank infrastructure is essential for the implementation of fast pipelines and novel techniques including single-cell and single-nucleus sequencing [refer to Freund et al. (2018) for a more detailed review]. Although novel rapid sequencing approaches integrated into patient biobanking are promising as they can generate data quickly, there are a substantial number of limitations including cost (specifically cost of large samples sizes for validation), lack of sensitivity for infrequent cell types and low abundance RNAs (specifically for single cell approaches), and the time required for high dimensional data analysis (Lahnemann et al., 2020).

## 2 Discussion

Despite advances in analytic techniques, neurologic biobanking and biomarker studies are limited by the inaccessibility of CNS tissues, cost and sustainability of robust biobanking efforts, and the fragility of the CNS microenvironment which supports intricate and dynamic cell–cell connections that are highly vulnerable to disruption. State-of-the-art biobanks, coupled with advanced technologies such as mass spectrometry, immunoassays, and NGS, will be crucial for the identification of novel biomarkers that may elucidate the molecular mechanisms of neurological disease, validate the importance of these markers in various pathologies, and develop the clinical utility of these markers. Given the expected increase in neurologic conditions among our aging population, the development of centralized state-of-the-art national [e.g., NIH NeuroBiobank (Freund et al., 2018)] and institutional [e.g., Barrow Neurological Institute (Seiler et al., 2015)] neurology-focused biobanks with integrated biomarker capabilities will be necessary to catalyze impactful translational neuroscience studies across disease states.

We developed an innovative and streamlined electronic consenting biobank, supported by a robust infrastructure and processing pipeline, for analysis of fresh specimens from individuals with neurological disorders. Importantly, developing innovative real-time biomarker analysis can enable earlier diagnosis, reliable prognostication, and invaluable insight into how a patient may respond to treatment modalities.

## 3 Conclusion

The development of neurological biobanks that are optimized for the collection of high-quality specimens and geared toward cross-institute collaboration is crucial for the discovery of diagnostic, surrogate and predictive biomarkers in neurological disease and their advancement toward clinical application. In the future, novel biomarkers may allow more accurate and accelerated diagnoses during the earliest stages of neurological disease (including the prodromal period), serve as surrogates of disease activity or progression thereby increasing the efficiency and speed of clinical trials, and predict therapeutic responsiveness to individual disease modifying therapies. Robust biobanks, supported by large and reliable clinical databases in addition to sustainable funding, will be essential for achieving those goals. In summary, construction of a high-quality, forward-thinking biobank connects neuroscientists with human specimens required for the identification, development, and validation of neurologic biomarkers.
